# Gaze Mapping and Observational Behavior During Nasal Endoscopy

**DOI:** 10.1002/alr.70145

**Published:** 2026-06-05

**Authors:** William S. Smithee, Duncan G. J. Green, Quinn F. O'Malley, Arjun Yusufji, Jonathan M. Owens, Edward D. McCoul

**Affiliations:** ^1^ Department of Otolaryngology–Head and Neck Surgery Tulane University New Orleans Louisiana USA; ^2^ Department of Otorhinolaryngology Ochsner Health New Orleans Louisiana USA; ^3^ Ochsner Clinical School University of Queensland New Orleans Louisiana USA

**Keywords:** eye tracking, gaze mapping, mucus, nasal endoscopy

## 1 Introduction

Nasal endoscopy (NE) is a cornerstone of the otolaryngologic examination. Guidelines identify direct visualization of mucopurulent drainage as a key diagnostic criterion for rhinosinusitis, emphasizing the clinician's ability to detect subtle findings within a narrow window of inspection [1, 2]. Growing evidence indicates that even experienced physicians may miss certain abnormalities, such as in mucus, due to cognitive bias, time constraints, or other pressures [3, 4].

**Key Points**

- Inter‐rater reliability on subtle nasal endoscopy findings, such as mucus, tends to be very low
- Gaze time was lower for mucus and other abnormal findings compared to normal anatomic features
- Most mucus findings fail to reach the gaze duration needed for perceptual recognition.

Across a range of clinical contexts, gaze‐mapping technology has been used to examine how experts visually interrogate images and operative fields, such as colonoscopy [5]. However, these dynamics have not been previously explored in the context of NE, where accurate image interpretation is central to diagnosis and surgical planning.

The present study investigates the relationship between gaze fixation time and diagnostic recognition, focusing on the detection of mucus. Our aim is to determine whether reporting an abnormal finding of mucus is associated with increased gaze fixation time on the corresponding area of interest (AOI).

## 2 Methods

Images were drawn from a de‐identified library of 100 NE examinations. Images included both normal anatomy and abnormalities. A total of 336 AOIs were delineated using Experiment Builder (SR Research). Each AOI corresponded to an anatomic region containing a structure or abnormality.

This was a prospective study evaluating visual attention during static NE using infrared eye tracking. Participants included medical students recruited from local institutions. Eligibility required the ability to view digital endoscopic images. Participation was voluntary and deemed exempt by the institutional review board of Ochsner Medical Center.

Participants were seated in front of a monitor equipped with the EyeLink Portable Duo system (SR Research, Toronto, Canada). Participants reviewed the set of 100 NE images. Each image contained one or more AOIs corresponding to normal anatomy (turbinate, sinus, choana, eustachian tube, and adenoid) or abnormal findings (mucus, mass, scar, and erythema).

Each image stimulus was displayed for 2000 ms, consistent with conventional exposure time used in eye‐tracking research [6]. Following each image, participants recorded whether the image was normal or abnormal and then selected specific features from a checklist.

Eye movements were recorded at 500 Hz using an EyeLink eye tracker (SR Research). The key extracted metric was gaze fixation time, the cumulative time duration spent within an AOI. Fixation was operationalized as ≥ 600 ms of gaze within an AOI, from a combined total of both eyes, consistent with the threshold for perceptual recognition as previously defined in the literature.

The primary outcome was the relationship between gaze fixation time on mucus AOIs and subsequent recognition of mucus as abnormal. Secondary outcomes included comparisons of gaze metrics between AOIs, the frequency of fixations meeting the 600 ms threshold across AOI types, and participant responses. Gaze fixation times were compared using t‐tests or one‐way ANOVA.

## 3 Results

Among 16 participants, 1029 (64.3%) of images were indicated as abnormal, with a median (IQR) gaze fixation time of 296 (0–928) ms.

**FIGURE 1:**
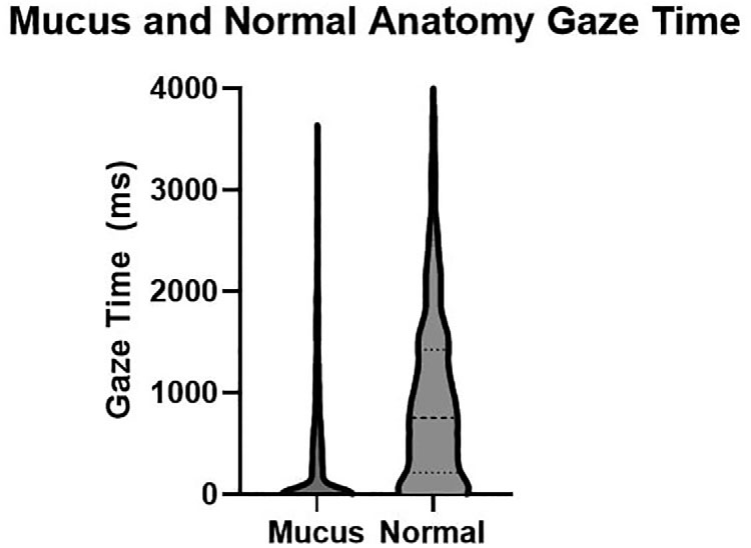
Violin plot of a *t*‐test of gaze fixation times between mucus AOIs and normal structure AOIs (*t* = 9.2, 1045 ms vs. 403.9 ms, *p* < 0.01).

**FIGURE 2:**
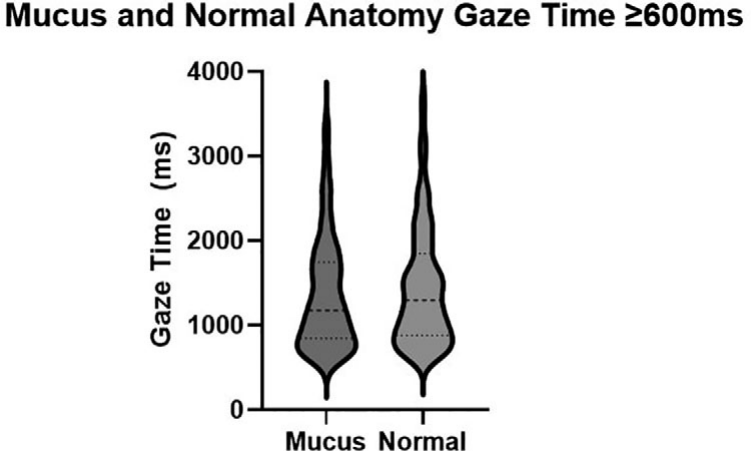
Violin plot of a t‐test of gaze fixation times between mucus AOIs and normal structure AOIs in instances where gaze fixation was at least 600 ms (*t* = 2.6, 1381 ms vs. 1643 ms, *p *< 0.05).

Mean (95% CI) gaze fixation time for mucus AOIs was 404 (361–447) ms compared to 1045 (974–1116) ms for normal nasal structure AOIs (*p* < 0.01) (Figure 1). The ≥ 600 ms threshold was less frequently recorded for mucus AOIs (22.7% [95% CI 16.3–29.1]) than for normal AOIs (57.4% [48.3–66.5], *p* < 0.01) (Figure 2). However, when < 600 ms trials were removed from both groups, the difference decreased (1381 [1017–1745] ms vs. 1643 [1396–1890] ms, *p* < 0.05).

Among trials with abnormalities, the mean gaze fixation time was not different compared to trials in which the participants indicated no abnormality (735 [555–915] ms vs. 711 [549–873] ms, *t* = 0.4, *p* = 0.63). Among cases with abnormalities, mean gaze fixation time was shorter for mucus (295 [146–444] ms) compared to non‐mucus abnormalities (612 [439–785] ms, *t* = 5.9, *p* < 0.01).

Across AOIs that included non‐mucus abnormalities (scar, mass, and erythema), the mean gaze fixation time of mucus AOIs (404 [361–447] ms) was shorter compared to all other abnormalities (876 [731–1021] ms, *p* < 0.01).

## 4 Discussion

Quantifying observational behavior using gaze mapping reveals that, under time constraints, relevant clinical features may remain unobserved. This was consistently demonstrated across AOIs of NE, but most prominently with mucus.

Inter‑rater reliability studies show that endoscopists agree on obvious findings such as polyps, but less well on mucosal edema [7]. This is supported by our findings, in which mean gaze times were significantly shorter for mucus than for normal structures. In addition, far fewer mucus observations met the 600 ms threshold compared to normal structures, suggesting a relative neglect of subtle abnormal findings.

These findings suggest a role for supplemental behavioral training. Gaze mapping can highlight instances where a trainee fails to achieve a settled gaze on mucus, even when that feature is visually present. Feedback could include highlighting of missed findings, providing annotated examples, measuring improvement, and adaptive training. Practitioners of NE should be reminded that perception itself is a potential source of error in diagnosis [8].

Several limitations bear mention. The stimuli were static images of brief duration, which are an artificial representation of input from NE in clinical practice. Participants may have required additional time to ascertain the orientation of the images and the structures within. Finally, the participants were medical students who lacked firsthand experience with NE. Recruitment of more experienced clinicians as subjects is an area for future study, though the effect may be difficult to predict given the low inter‐rater agreement of subtle findings [9].

## Funding

This work was supported by grants from Ochsner Health (INVEST‑MS 2023) and the Eye, Ear, Nose and Throat Foundation (EE231102).

## Conflicts of Interest

Edward D. McCoul serves as a consultant for 3D Matrix, Advanced Rx, Sanofi/Regeneron, and Zsquare. All other authors declare no conflicts of interest.
